# Distinct [^18^F]THK5351 binding patterns in primary progressive aphasia variants

**DOI:** 10.1007/s00259-018-4075-3

**Published:** 2018-06-26

**Authors:** Jolien Schaeverbeke, Charlotte Evenepoel, Lieven Declercq, Silvy Gabel, Karen Meersmans, Rose Bruffaerts, Kate Adamczuk, Eva Dries, Karen Van Bouwel, Anne Sieben, Yolande Pijnenburg, Ronald Peeters, Guy Bormans, Koen Van Laere, Michel Koole, Patrick Dupont, Rik Vandenberghe

**Affiliations:** 1 Laboratory for Cognitive Neurology, Department of Neurosciences, KU Leuven, Herestraat 49, 3000 Leuven, Belgium; 2 Alzheimer Research Centre KU Leuven, Leuven Research Institute for Neuroscience & Disease, KU Leuven, Herestraat 49, 3000 Leuven, Belgium; 3 Laboratory of Radiopharmaceutical Research, KU Leuven, Herestraat 49, 3000 Leuven, Belgium; 4 Nuclear Medicine and Molecular Imaging, University Hospitals Leuven, Herestraat 49, 3000 Leuven, Belgium; 5 Neurology Department, University Hospitals Leuven, Herestraat 49, box 7003, 3000 Leuven, Belgium; 6 Neurodegenerative Brain Diseases Group, Center for Molecular Neurology, VIB, Universiteitsplein 1, 2610 Antwerp, Belgium; 7 Institute Born-Bunge, Neuropathology and Laboratory of Neurochemistry and Behavior, University of Antwerp, Universiteitsplein 1, 2610 Antwerp, Belgium; 8 Neurology Department, University Hospital Ghent, Corneel Heymanslaan 10, 9000 Ghent, Belgium; 9 Old Age Psychiatry Department, GGZinGeest, Van Hilligaertstraat 21, 1072 JX Amsterdam, The Netherlands; 10 Alzheimer Center & Department of Neurology, VU University Medical Center, De Boelelaan 1117, 1081 HV Amsterdam, The Netherlands; 11 Radiology Department, University Hospitals Leuven, Leuven, Belgium

**Keywords:** [^18^F]THK5351 binding, Primary progressive aphasia, Tau, Nonfluent variant, Motor speech, Agrammatism

## Abstract

**Purpose:**

To assess the binding of the PET tracer [^18^F]THK5351 in patients with different primary progressive aphasia (PPA) variants and its correlation with clinical deficits. The majority of patients with nonfluent variant (NFV) and logopenic variant (LV) PPA have underlying tauopathy of the frontotemporal lobar or Alzheimer disease type, respectively, while patients with the semantic variant (SV) have predominantly transactive response DNA binding protein 43-kDa pathology.

**Methods:**

The study included 20 PPA patients consecutively recruited through a memory clinic (12 NFV, 5 SV, 3 LV), and 20 healthy controls. All participants received an extensive neurolinguistic assessment, magnetic resonance imaging and amyloid biomarker tests. [^18^F]THK5351 binding patterns were assessed on standardized uptake value ratio (SUVR) images with the cerebellar grey matter as the reference using statistical parametric mapping. Whole-brain voxel-wise regression analysis was performed to evaluate the association between [^18^F]THK5351 SUVR images and neurolinguistic scores. Analyses were performed with and without partial volume correction.

**Results:**

Patients with NFV showed increased binding in the supplementary motor area, left premotor cortex, thalamus, basal ganglia and midbrain compared with controls and patients with SV. Patients with SV had increased binding in the temporal lobes bilaterally and in the right ventromedial frontal cortex compared with controls and patients with NFV. The whole-brain voxel-wise regression analysis revealed a correlation between agrammatism and motor speech impairment, and [^18^F]THK5351 binding in the left supplementary motor area and left postcentral gyrus. Analysis of [^18^F]THK5351 scans without partial volume correction revealed similar results**.**

**Conclusion:**

[^18^F]THK5351 imaging shows a topography closely matching the anatomical distribution of predicted underlying pathology characteristic of NFV and SV PPA. [^18^F]THK5351 binding correlates with the severity of clinical impairment.

**Electronic supplementary material:**

The online version of this article (10.1007/s00259-018-4075-3) contains supplementary material, which is available to authorized users.

## Introduction

Primary progressive aphasia (PPA) is a neurodegenerative syndrome which primarily affects speech and language, with relative preservation of other cognitive domains [1]. Current consensus recommendations describe clinical criteria for three subtypes: a nonfluent/agrammatic variant (NFV), a semantic variant (SV), and a logopenic variant (LV) [1]. Patients with NFV PPA present with agrammatism and/or speech apraxia, whereas patients with SV PPA have single-word comprehension and naming deficits [1]. Patients with LV PPA show deficient single-word retrieval in spontaneous speech and a short-term phonological memory deficit [1]. Of patients with NFV, 50–70% have underlying frontotemporal lobar degeneration (FTLD) tauopathy [2–4]. FTLD tauopathy can be four-repeat (4R) tau due to corticobasal degeneration (CBD) or progressive supranuclear palsy (PSP), which is localized in the basal ganglia, brainstem and cerebral cortex [5], or, less frequently, 3R tau Pick’s disease pathology [2, 3]. Approximately 25% of patients with NFV PPA have FTLD transactive response DNA binding protein 43-kDa (TDP-43) pathology, usually of type A [2], and 12–25% have Alzheimer disease (AD) pathology [3, 4] (for review see [6]), characterized by neurofibrillary tangles composed of balanced 3R/4R tau, and fibrillar amyloid plaques. Of patients with SV PPA, 69–83% have FTLD TDP-43 type C pathology in the temporal cortex bilaterally [2–4, 7] and 10–33% have underlying AD pathology [3, 4]. Pick’s disease can also cause SV PPA [2, 3, 7]. Of patients with LV PPA, 56–100% have underlying AD pathology [2–4]. In LV PPA, the superior temporal gyrus and inferior parietal lobule are affected asymmetrically with predominance of the neurofibrillary tangle load in the left hemisphere [4].

Although there is some concordance between each pathology and its clinical presentation, the clinical diagnosis does not provide a reliable indication of the underlying pathology in the individual PPA patient [3]. For instance, although the likelihood of a primary FTLD tauopathy is higher in patients with NFV PPA, AD pathology may also be a cause [3, 4]. Moreover, in many patients, a tauopathy cannot be reliably distinguished from a TDP-43 proteinopathy as the underlying cause based on clinical grounds [3, 4]. The ability to discriminate between these two distinct causes in vivo is essential for the development of disease-modifying therapies. Furthermore, molecular targets, which can serve as noninvasive markers of drug target engagement and disease progression, are needed for clinical drug development.

The recent introduction of tau PET ligands, including [^18^F]AV1451 (T807 or flortaucipir) [8] and [^18^F]THK5351 [9], may create the opportunity to measure tauopathy in vivo. [^18^F]AV1451 binds robustly to AD-related tauopathy, while binding to 4R tau is inconclusive [10–14]. By contrast, [^18^F]THK5351 shows more prominent binding in FTLD (CBD and PSP) than in AD tauopathy [9, 15–20]. In PPA patients, [^18^F]AV1451 has been demonstrated to be able to discriminate among the three subtypes [21]. A principal components analysis has shown that two components allow reliable discrimination: the degree of increase and the frontotemporal gradient. Standardized uptake value ratios (SUVR) were found to be higher in patients with LV PPA than in those with the two other variants, suggesting that this ligand probably has higher affinity for AD tauopathy than for FTLD tauopathy. In patients with SV PPA, binding was mainly situated in the anterior temporal cortex while in patients with NFV PPA, [^18^F]AV1451 binding was principally seen in the frontal white matter and subcortical nuclei [21]. The discriminative ability of [^18^F]AV1451 was as high as that of [^18^F]FDG PET and higher than that of MRI [21].

There have also been a number of smaller case studies investigating the use of PET for determining tau burden in patients with PPA, some of them including neuropathological confirmation [12]. In a patient with NFV PPA and in a patient with PSP with nonfluent aphasia, elevated [^18^F]THK5351 binding was seen in the frontal cortex [18, 19]. In a patient with SV PPA, binding of both [^18^F]AV1451 [21–23] and [^18^F]THK5351 [19, 24, 25] in the anteroinferior and lateral temporal cortices has been reported. This is surprising as SV is mainly a TPD-43 proteinopathy. Elevated binding of [^18^F]AV1451 [19, 21, 26–29] and [^18^F]THK5351 has been demonstrated in the posterior temporal cortex and inferior parietal lobule bilaterally in single patients with LV PPA [17, 19], which mirrors the [^18^F]FDG binding pattern [17].

The primary objective of this study was to evaluate the ability of [^18^F]THK5351 to differentiate different PPA variants known to have different probabilities of underlying neuropathology. As yet, no study has directly compared [^18^F]THK5351 binding among all three PPA variants. Building on previous evidence, we hypothesized that [^18^F]THK5351 would show a positive signal in disease-specific regions in all PPA variants, with a regionally specific pattern for each variant. The secondary objective was to assess the association between [^18^F]THK5351 binding and clinical measures of speech and language deficits. Elevated [^18^F]THK5351 binding was predicted to correlate with clinical measures of language and speech deficits based on the notion that tau levels on PET generally correlate well with cognition [26, 27]. As yet such a correlation has not been demonstrated for speech and language measures in patients with PPA.

## Materials and methods

### Study participants

A consecutive series of 21 patients who fulfilled the international consensus criteria for PPA [1] were enrolled between August 2016 and October 2017. Of these 21 patients, 18 were recruited through the memory clinic of the University Hospitals Leuven, one (patient 3) was referred to the study from the Free University Amsterdam and two (patients 17 and 20) from the University Hospitals Ghent (Table 1). One patient (patient 15) had to be excluded due to a subarachnoid cyst anterior to the left temporal lobe. The patients were classified on the basis of the clinical evaluation by an experienced neurologist in combination with the results of clinical MRI and [^18^F]FDG PET scans. In a subset of 13 PPA patients AD biomarkers were measured in the cerebrospinal fluid (CSF) as part of the clinical work-up. This was performed by the Laboratory of Medical Analysis, Medicine Department of UZ Leuven, using Innotest ELISA for amyloid-β_42_ (Aβ_1–42_; cut-off 853 pg/ml [32]), total tau (t-tau; Aβ_1–42_/t-tau cut-off 2.258), and phospho_181_-tau (p_181_-tau; Fujirebio Europe, Ghent, Belgium) (Table 1). Two patients (patients 9 and 14) received ^11^C-labelled Pittsburgh compound B ([^11^C]PIB) PET as part of the prior clinical work-up [33] (Table 1). In PPA patients in whom amyloid biomarkers had not been measured, [^11^C]PIB PET was acquired for the current study. Of the 20 PPA patients included, 12 fulfilled the consensus criteria for NFV, 5 for SV, and 3 for LV PPA. Clinical signs and symptoms associated with PSP or CBD were documented on neurological/clinical examination (Supplementary Table 1).

**Table 1:** Demographics, neurolinguistic and neuropsychological assessment of the 20 included PPA patients

	Patient number																			
	2	3	4	12	13	14	16	17	18	19	20	21	1	5	6	8	10	7	9	11
PPA variant	NFV	NFV	NFV	NFV	NFV	NFV	NFV	NFV	NFV	NFV	NFV	NFV	SV	SV	SV	SV	SV	LV	LV	LV
Age (years)	80	57	62	68	76	66	70	65	49	63	70	76	73	71	63	52	55	77	63	74
Gender	M	F	F	F	M	F	M	M	F	M	M	F	F	F	F	F	M	M	F	M
Education (years)	17	16	12	10	12	15	12	15	12	10	10	12	14	8	13	13	14	10	12	18
Handedness	R	R	L	R	R	R	R	R	R	R	R	R	R	R	R	R	R	R	R	R
Symptom duration (months)	33	43	16	60	37	74	39	40	53	29	45	24	19	44	11	131	6	59	95	48
[^11^C]PIB SUVR	NC	NC	1.26	NC	2.1	1.16	1.35	1.57	NC	1.37	NC	NC	NC	NC	1.12	1.14	1.2	NC	1.81	NC
CSF Aβ_42_ (pg/ml)	816	1,057	NC	832	477	NC	NC	759	1,077	NC	887	1,144	1,558	733	NC	NC	NC	564	664	321
CSF t-tau (pg/ml)	195	247	NC	320	442	NC	NC	744	231	NC	270	265	428	262	NC	NC	NC	407	NC	858
CSF Aβ_42_/t-tau	4.18	4.28	NC	2.60	1.08	NC	NC	1.02	4.66	NC	3.29	4.32	3.64	2.80	NC	NC	NC	1.39	NC	0.37
CSF p_181_-tau (pg/ml)	42	34	NC	43	59.6	NC	NC	87.1	31	NC	48	39.9	52	36.3	NC	NC	NC	65.7	NC	95
Amyloid-positivity	−	−	−	−	+	−	−	+	−	−	−	−	−	−	−	−	−	+	+	+
CDR	1	0.5	0.5	0.5	0.5	0	0.5	1	NC	0.5	0.5	0.50	1	0.5	0.5	0.5	0.5	1	0	0.5
MMSE score (/30)	28	**24**	28	**18**	**24**	30	**23**	**5**	NC	**26**	**26**	**18**	**26**	**25**	29	**23**	**30**	**26**	28	**24**
CPM score (/36)	**26**	NC	31	29	29	**26**	29	**24**	NC	**25**	26	**17**	28	27	34	36	34	32	32	**12**
BNT score (/60)	52	**7**	**46**	**30**	**43**	47	**41**	**4**	**17**	53	53	48	**11**	**14**	**17**	**9**	**33**	46	57	**24**
AVF score (1 min)	**8**	**7**	**12**	**2**	**7**	**13**	**9**	**2**	NC	16	**4**	**5**	**7**	**11**	**13**	**6**	16	**7**	23	**8**
AAT sum single-word comprehension score (/60)	**41**	56	**49**	55	**41**	51	**42**	**37**	**27**	53	51	**47**	**31**	**32**	**39**	**35**	58	53	54	**50**
PALPA auditory word–picture matching score (/40)	40	**37**	39	39	39	**38**	39	**38**	NC	40	40	**36**	**26**	**27**	**26**	**21**	39	**38**	39	**38**
PALPA verbal associative-semantic high imageability (/15)	**11**	15	15	**10**	**12**	14	14	**11**	NC	13	**10**	14	**9**	15	**12**	**5**	14	15	15	15
PALPA verbal associative-semantic low imageability (/15)	12	14	**11**	**7**	14	12	**11**	**7**	NC	**11**	**9**	**5**	**3**	**7**	**10**	**6**	14	13	14	12
PPT score (/52)	**46**	**47**	**47**	**48**	**47**	**48**	**47**	**47**	NC	49	**48**	**45**	**31**	**31**	**38**	**34**	**47**	49	52	**45**
BORB easy B score (/32)	28	31	**25**	30	29	28	28	29	NC	30	28	27	**22**	**19**	**22**	**18**	**25**	28	30	**26**
BORB hard A score (/32)	25	31	**19**	22	22	26	24	**20**	NC	**21**	30	**20**	**17**	**19**	**22**	**17**	26	23	26	**21**
WEZT verb comprehension score (/60)	**56**	60	**45**	**40**	**55**	**42**	**52**	**42**	NC	58	**55**	**49**	**43**	**32**	**51**	**48**	58	57	57	**53**
WEZT auditory sentence comprehension score (/40)	**36**	**29**	**36**	**26**	**33**	**35**	**31**	**12**	NC	**33**	**32**	**23**	**37**	**33**	38	38	39	**27**	40	**37**
WEZT active sentence anagram score (/10)	10	10	10	**9**	**9**	10	10	**5**	NC	10	10	10	10	10	10	10	10	10	10	10
WEZT passive sentence anagram score (/10)	10	10	**9**	**6**	**9**	10	**5**	**5**	NC	10	**3**	**5**	10	**9**	10	**9**	10	10	10	10
AAT cognate word repetition score (/30)	29	**28**	30	30	**16**	30	29	NC	**5**	**29**	**27**	30	30	30	**28**	30	30	30	**29**	29
AAT sentence repetition score (/30)	**27**	**17**	28	**24**	28	28	28	NC	**0**	**25**	**23**	**27**	29	29	30	**27**	30	**13**	**26**	**24**
PALPA single word repetition score (/80)	77	76	80	79	**55**	80	77	NC	NC	**49**	76	80	79	77	77	80	79	79	80	80
PALPA pseudoword repetition score (/80)	**21**	63	72	72	**11**	78	57	NC	NC	**20**	59	**53**	77	69	67	79	77	66	74	**56**
DIAS diadochokinesis score	103	**24**	**50**	**51**	**42**	77	115	75	NC	79	**18**	**32**	**50**	70	147	125	80	77	114	117

Values in bold are significantly different from those in healthy controls based on Crawford and Garthwaite [30] or Crawford and Howell [31] statistical procedures

*AAT* Akense Afasie Test (Aachen Aphasia Test), *Aβ*_*1–42*_ amyloid-β_42_, *AVF* Animal Verbal Fluency, *BNT* Boston Naming Test, *BORB* Birmingham Object Recognition Battery, *CDR* Clinical Dementia Rating scale, *[*^*11*^*C]PIB* ^11^C-Pittsburgh Compound B, *CPM* Coloured Progressive Matrices, *CSF* cerebrospinal fluid, *DIAS* Diagnostisch Instrument voor Apraxie van de Spraak (Diagnostic Instrument for Apraxia of Speech), *LV* logopenic variant, *MMSE* Mini-Mental State Examination, *NFV* nonfluent variant, *PALPA* Psycholinguistic Assessment of Language Processing in Aphasia, *PPA* primary progressive aphasia, *PPT* Pyramids and Palm Trees Test, *p181-tau* phospho181-tau, *SUVR* standardized uptake value ratio in a composite cortical volume of interest, *SV* semantic variant, *WEZT* Werkwoorden En Zinnen Test. *NC* no data collected

For normative reasons, we recruited 23 cognitively intact older healthy controls matched for age (Kruskall Wallis *H*(3) = 2.38, *P* = 0.50), education (*H*(3) = 1.20, *P* = 0.75) and gender (*χ*^2^(3) = 1.97, *P* = 0.58) with the group of PPA patients through advertisements in newspapers and online. Inclusion criteria were a Mini-Mental State Examination (MMSE) score of ≥27, a Clinical Dementia Rating scale (CDR) global score of zero and neuropsychological test scores within 1.9 of the standard deviations of norms adjusted for age, gender and education [33]. Healthy controls had no history of neurological or psychiatric disease or any brain lesions on structural MRI. White matter lesions were not an exclusion criterion.

The study protocol (EudraCT 2014-002976-10) was approved by the UZ/KU Leuven Ethics Committee for Research. All participants provided written informed consent in accordance with the principles of the Declaration of Helsinki after receiving a complete description of the study protocol.

### Neuropsychological and neurolinguistic protocol

All study participants underwent a standard neuropsychological and neurolinguistic examination. General cognitive functioning was assessed using the CDR and MMSE. Nonverbal fluid intelligence was assessed using Coloured Progressive Matrices (CPM). Confrontation naming was assessed using the Boston Naming Test (BNT) and category verbal fluency using the 1-min Animal Verbal Fluency (AVF) test. Single-word comprehension (spoken and written input modality) was assessed using the Dutch versions of the Aachen Aphasia Test (*Akense Afasie Test*, AAT) and associative-semantic ability was assessed using the Psycholinguistic Assessment of Language Processing in Aphasia (PALPA; subtest 45). PALPA subtest 49 and the picture version of the Pyramids and Palm Trees Test (PPT). Object identification was assessed using the Birmingham Object Recognition Battery (BORB), easy (B) and hard (A). Sentence comprehension and grammaticality were assessed using the *Werkwoorden En Zinnen Test* (WEZT) [34] (verbs and sentences test). Repetition was assessed using the AAT repetition test and real words and pseudowords using the PALPA subtest 9. Speech apraxia was assessed using the *Diagnostisch Instrument voor Apraxie van de Spraak* (DIAS) [35] (Diagnostic Instrument for Apraxia of Speech). The DIAS allows the examiner to assess consonant and vowel repetition (DIAS severity score). The DIAS also includes a diadochokinesis task, which assesses the ability to make antagonistic movements using different parts of the mouth, tongue and soft palate in quick succession.

The scores of individual PPA patients were compared against those of a larger group of 67 cognitively intact older controls, including 23 control subjects who participated in the PET study, to increase the statistical power for comparisons with PPA patients. These 67 controls were matched for age (*H*(3) = 2.33, *P* = 0.51), education (*H*(3) = 1.48, *P* = 0.69) and gender (*χ*^2^(3) = 1.987, *P* = 0.58) with the group of PPA patients and fulfilled the same inclusion and exclusion criteria described in section Study participants.

### [^18^F]THK5351 PET acquisition and analysis

[^18^F]THK5351 PET scans were acquired on a 16-slice Siemens Biograph PET/computed tomography (CT) scanner (Siemens Medical Solutions, Erlangen, Germany) in 20 patients and in 20 of the 23 healthy control subjects (three control subjects withdrew from the study due to claustrophobia). After bolus injection of [^18^F]THK5351 (mean dose 185.2 MBq, range 178.7–191.0 MBq) into an antecubital vein, five healthy control subjects were scanned dynamically with arterial sampling between 0 and 100 min after injection to determine the optimal PET imaging window. The remaining healthy controls and all PPA patients were scanned between 50 and 80 min after injection of [^18^F]THK5351 (mean dose 184.1 MBq, range 165.8–196.0 MBq, in controls; mean dose 181.8 MBq, range 164.9–192.3 MBq, in patients). A low-dose CT scan was acquired for attenuation correction prior to the PET scan. PET emission images were acquired in 3D list mode and subsequently reconstructed as six 5-min frames using the ordered subsets expectation maximization algorithm (four iterations, 16 subsets) [32]. [^18^F]THK5351 PET emission frames were realigned to correct for head motion, summed and rigidly coregistered to the subject’s T_1_-weighted MRI scan using statistical parametric mapping software (SPM12; Wellcome Trust Centre for Neuroimaging, London, UK) implemented in Matlab R2014b (Mathworks, Natick, MA). The MRI scan was segmented using SPM12 into grey matter, white matter and CSF. The summed PET images were subsequently corrected for partial volume effects (partial volume correction, PVC) using the modified method of Müller-Gärtner et al. [36]. All images were then warped to MNI template space using the deformation field obtained during the segmentation step. [^18^F]THK5351 SUVR images with PVC were subsequently created using the subject-specific cerebellar grey matter as reference region. [^18^F]THK5351 SUVR images were also calculated without PVC. For voxel-based statistical analyses, [^18^F]THK5351 SUVR images were smoothed with an isotropic 8-mm full-width at half-maximum (FWHM) gaussian kernel.

#### Quantification of [^18^F]THK5351 PET binding per PPA variant

An approach similar to that used by Josephs et al. [21] was used to compare the regional effect sizes between variants. Regions of interest (ROIs) were selected from the Automated Anatomical Labeling (AAL) atlas [37] based on their typical involvement in the different variants of PPA [21]. In addition to the regions used by Josephs et al. [21] a medial temporal region (hippocampus, parahippocampus and amygdala) and a medial parietal region (posterior cingulum and precuneus) were also included. The binary ROIs were made subject-specific by intersecting them with the individual’s grey matter map, thresholded at 0.3 [32]. The mean [^18^F]THK5351 SUVR was calculated for each PPA patient in all subject-specific ROIs per hemisphere.

### Amyloid biomarker measurement and analysis

[^11^C]PIB PET scans were processed in SPM12 using the same MRI-based method as described for [^18^F]THK5351. The mean [^11^C]PIB PET SUVR was calculated in a neocortical composite region [32] and the [^11^C]PIB PET scan was considered positive if this value was significantly higher than that in healthy controls based on a modified *t *test (*α* < 0.05) [31].

### Volumetric MRI acquisition and analysis

A high resolution T_1_-weighted structural MRI scan was acquired on the same day as the neuropsychological testing and used for processing the PET data. As a secondary analysis voxel-based morphometry (VBM8) [38] was performed on the T_1_-weighted images as previously described [33]. For voxel-based statistical analyses, modulated grey matter maps were smoothed with an 8-mm FWHM gaussian 3D kernel.

### Statistical analysis

Standard statistical analyses were performed using SPSS, version 24 (IBM Statistics, Armonk, NY). The significance threshold was set at *α* < 0.05 for all standard statistical analyses. Demographic and neuropsychological data were statistically compared between groups using the two-tailed Kruskal-Wallis and two-tailed post hoc Mann-Whitney *U* tests for continuous variables and the two-tailed Pearson chi-squared test for categorical variables. Graphics were prepared with Matlab2014b and MRIcron.

#### Primary outcome analysis

To assess [^18^F]THK5351 binding patterns at the group level, a voxel-wise analysis of variance (ANOVA) was performed in SPM12 implemented in Matlab R2014b with [^18^F]THK5351 SUVR images as within-subjects factor and diagnostic groups (healthy controls, NFV, SV and LV PPA) as between-subjects factor. This analysis was performed with and without PVC. The default significance threshold for this analysis was set at voxel-level uncorrected *P* < 0.001 combined with a cluster-level family-wise error-corrected threshold *P* < 0.05 [39]. At the individual patient level, [^18^F]THK5351 SUVR images with PVC were compared with the mean and standard deviation [^18^F]THK5351 SUVR images with PVC of the healthy control group (*n* = 20) using a voxel-wise modified *t* test [31] developed using in-house software. The threshold was set at voxel-level uncorrected *P* < 0.001.

#### Secondary outcome analyses

##### [^18^F]THK5351 binding and clinical impairment

To reveal the underlying structure and to reduce the dimensionality of the neurolinguistic dataset, a factor analysis was performed in SPSS. PPA patient 18 was excluded because only limited neuropsychological data were available as a result of disease severity. Two healthy control subjects were also excluded because data were missing. Hence, the data from 40 subjects (19 patients and 21 healthy controls) were available for factor analysis (Kaiser-Meyer-Olkin’s test score of 0.77 and Bartlett’s test *P* < 0.001). The principal axis factoring method was applied to extract factors with an eigenvalue of >1.0 (Kaiser’s criterion). The eigenvalue of each factor corresponds to the proportion of variance explained by that factor. The factors were rotated with a VARIMAX orthogonal rotation to obtain interpretable and uncorrelated factor loadings. Only factor loadings greater than 0.7 were retained [40]. In a next step, individual weighted factor scores were calculated for each extracted factor using a regression-based approach. Weighting refers here to the factor loading of that variable, i.e. the higher the factor loading of a variable, the more that variable is associated with the underlying factor. Missing values were replaced by the mean score from the entire group for that variable.

In a next step, a whole-brain voxel-wise linear regression analysis across PPA patients was performed for each extracted factor separately, with the individual weighted factor scores as the independent variable and the [^18^F]THK5351 PVC SUVR images as dependent variable, corrected for education, age and gender. Only individual weighted factor scores that were not a priori classified by Grubb’s test as significant outliers (*α* < 0.05) were included in the whole-brain voxel-wise regression analysis. As Grubb’s test classified the weighted factor 2 score of patient 17 as a significant outlier, this patient was excluded from the regression analysis. The significance threshold was a voxel level uncorrected *P* < 0.001 combined with a cluster level family-wise error correction at *P* < 0.05 [39].

For the sake of completeness, whole-brain voxel-wise linear regression analyses across PPA patients were also performed for the individual tests used in the factor analysis (after outlier exclusion). For each significant cluster, the mean voxel value in that cluster was extracted for each individual PPA patient to visualize the slope of the correlation between the variables solely for illustrative purposes.

##### Topography of [^18^F]THK5351 binding and atrophy

To compare the pattern of [^18^F]THK5351 binding with the pattern of atrophy, a voxel-wise ANOVA was performed with modulated grey matter images as within-subjects factor and diagnostic groups as between-subjects factor, similar to the procedure described in section Primary outcome analysis.

## Results

Patients with the different PPA variants did not differ in terms of age (*H*(2) = 1.79, *P* = 0.41), education (*H*(2) = 0.062, *P* = 0.97), gender (*χ*^2^(2) = 1.95, *P* = 0.38) or symptom duration (*H*(2) = 4.00, *P* = 0.14). Two of 12 NFV patients (patient 13, 76 years old, and patient 17, 65 years old), all of the LV patients and none of the SV patients were amyloid-positive (Table 1).

### Primary outcome analysis

#### Voxel-level statistical analysis of [^18^F]THK5351 binding in PPA variants

**Fig. 1:**
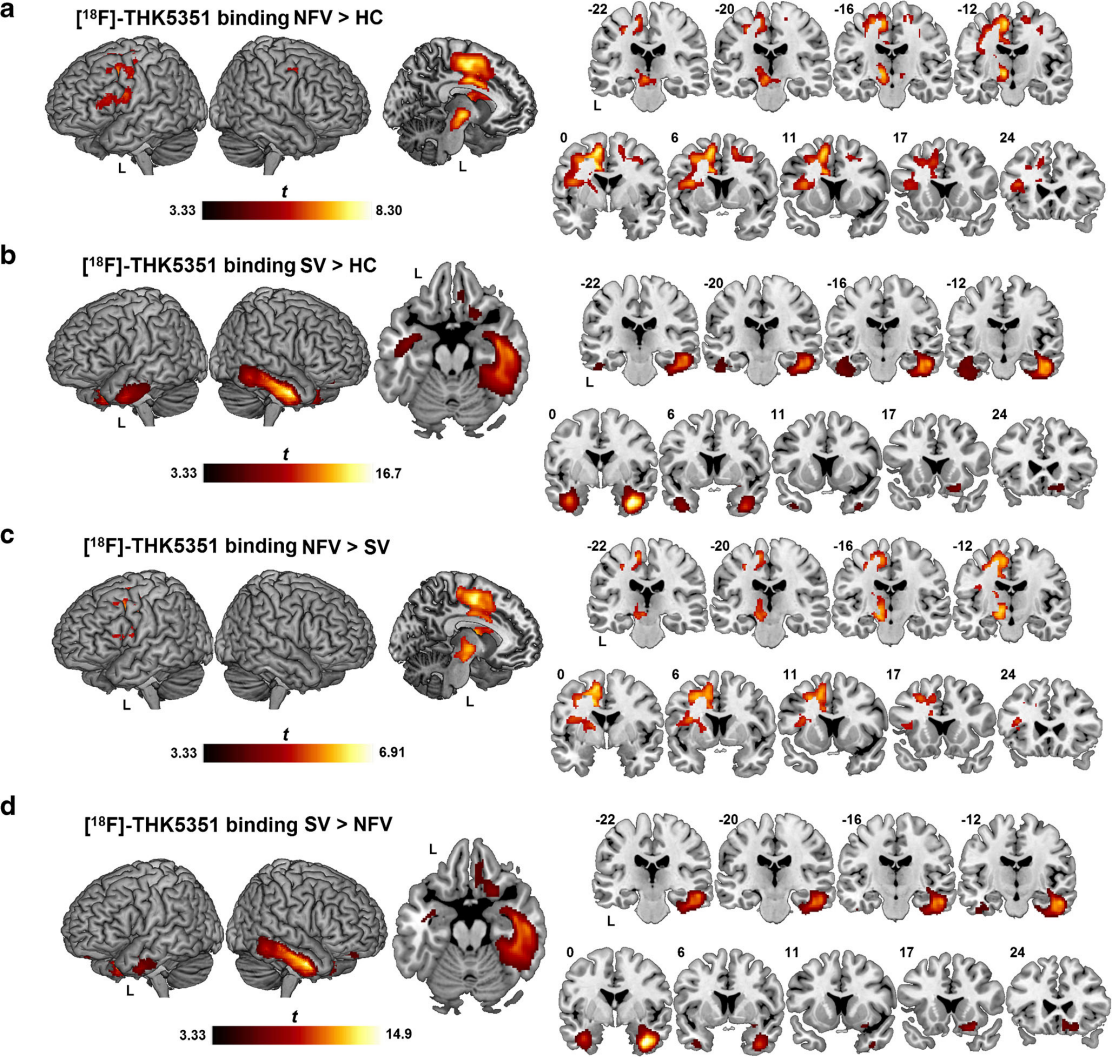
[^18^F]THK5351 binding pattern in PPA variants. Significantly elevated [^18^F]THK5351 binding on SUVR images with partial volume correction compared using voxel-wise ANOVA is depicted as a *t*-contrast overlaid on MNI template brain renderings and on coronal slices (brighter colour means higher *t* value). Higher binding in (**a**) nonfluent variant (NFV) PPA patients and (**b**) semantic variant (SV) PPA patients compared with healthy controls (HC), (**c**) NFV patients compared with SV patients, and (**d**) SV patients compared with NFV patients. The significance threshold was set at voxel-level uncorrected *P* < 0.001 combined with cluster-level family-wice error-corrected threshold *P* < 0.05

NFV patients showed increased [^18^F]THK5351 binding in the supplementary motor area, precentral gyrus and premotor cortex bilaterally, in the left cingulum, pars triangularis, pars opercularis, insula, basal ganglia, thalamus, and in the midbrain nuclei (subthalamic nucleus, red nucleus and substantia nigra) compared with controls in the PVC-based analysis (Fig. 1a; Table 2). SV patients showed significantly increased [^18^F]THK5351 binding in the temporal lobes bilaterally (right > left), including the temporal pole, inferior and middle temporal gyri, amygdala, anterior hippocampus, parahippocampal gyrus and entorhinal cortex, and in the fusiform gyri and the right ventromedial frontal cortex compared with controls (Fig. 1b; Table 2). LV patients did not show elevated binding compared with controls, most probably because of the small sample size and the heterogeneity among the individual patterns. Similar results were obtained without PVC for NFV and SV patients compared with controls, although the clusters of elevated [^18^F]THK5351 binding in the left hemisphere of NFV patients appeared larger than with PVC (Supplementary Fig. 1a) and an additional cluster was obtained in the left ventromedial frontal cortex in SV patients (Supplementary Fig. 1b).

**Table 2:** Peak coordinates of whole-brain voxel-wise ANOVA with PVC of [^18^F]THK5351 binding in PPA

Cluster			Peak		MNI coordinates		
Regions of [^18^F]-THK5351 binding	*P* value	Size	*T*	*Z*	*x*	*y*	*z*
NFV patients > healthy controls							
Supplementary motor area,	<0.001	5,353	8.30	6.17	−16	−4	48
Premotor cortex,			7.10	5.58	−12	−14	−4
Basal ganglia,			6.85	5.44	−36	−6	44
Midbrain,	0.001	626	5.39	4.59	16	4	54
Thalamus			4.91	4.27	16	−16	60
			4.84	4.22	12	−8	60
SV patients > healthy controls							
Temporal lobes,	<0.001	5,063	16.67	65,535	38	0	−32
Right ventromedial frontal			13.19	65,535	52	−26	−14
cortex			12.80	7.80	52	−14	
	<0.001	1,684	11.94	7.55	−36	−2	−30
	0.025	331	6.25	5.11	26	16	−16
			4.07	3.67	8	28	−12
			4.01	3.62	8	38	−14
NFV patients > SV patients							
Supplementary motor area,	<0.001	2,354	6.91	5.48	−16	−4	48
Premotor cortex,				5.62	4.73	−18	−10
Basal ganglia,			5.48	4.64	−26	4	
Midbrain,	<0.001	1,405	5.66	4.75	−12	−12	−4
Thalamus			5.32	4.54	−20	−8	16
			4.77	4.17	−36	4	18
SV patients > NFV patients							
Temporal lobes,	<0.001	5,445	14.91	65,535	36	0	−34
Right ventromedial frontal			11.67	7.46	52	−26	−14
cortex			11.40	7.37	52	−12	−24
	0.001	711	9.01	6.48	−36	−2	−30

The significance threshold was set at voxel-level uncorrected *P* < 0.001 combined with cluster-level family-wise error-corrected threshold *P* < 0.05

*NFV* nonfluent variant, *SV* semantic variant, *T* *t* value, *Z* *z* value

The pattern of increased binding of [^18^F]THK5351 in the left-hemispheric regions in NFV patients compared with SV patients was similar to that seen for the comparison between NFV patients and controls, with the highest binding in the supplementary motor area (Fig. 1c; Table 2). The pattern of increased [^18^F]THK5351 binding in SV patients was similar to that seen for the comparison between SV patients and controls, with the highest binding in the right fusiform gyrus and the right inferior and middle temporal gyri (Fig. 1d). Binding in the right ventromedial frontal cortex was also increased in SV patients compared with NFV patients (Fig. 1d; Table 2). The results of between-group comparisons without PVC showing increased binding in NFV and SV patients were similar to those with PVC (Supplementary Fig. 1c, d).

**Fig. 2:**
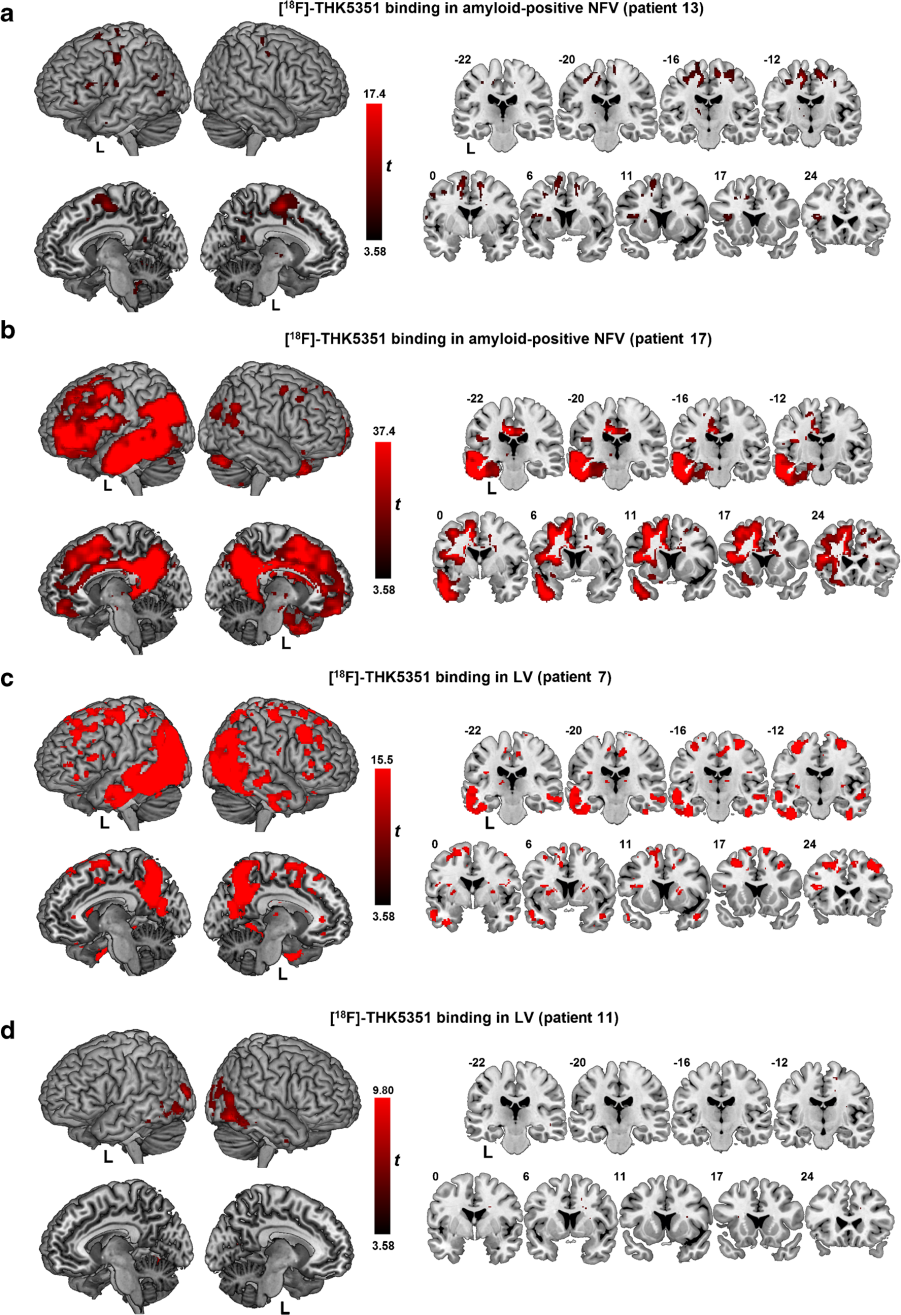
[^18^F]THK5351 binding pattern in amyloid-positive NFV patients and in two LV patients. Significantly elevated [^18^F]THK5351 binding on SUVR images with partial volume correction compared with that in healthy controls using a voxel-wise *t* test modified according to the method of Crawford in individual patients: **a** nonfluent variant (NFV) patient 13, **b** NFV patient 17, **c** logopenic variant (LV) patient 7, **d** LV patient 11. *T*-contrasts are overlaid on MNI template brain renderings and on coronal slices (brighter colour means higher *t* value). The significance threshold was set at voxel-level uncorrected *P* < 0.001 for the Crawford *t*-contrasts. Patient numbers refer to Table 1

At the individual patient level, of 12 NFV patients, 8 showed significantly increased binding in the left premotor cortex, 9 in the supplementary motor area, 8 in the midbrain and 5 in the basal ganglia. One NFV patient also showed increased binding in the left frontal and temporal cortical regions. The amyloid-positive NFV patient (patient 13) showed increased binding in the supplementary motor area and the dorsal premotor cortex (Fig. 2a). NFV patient 17 also showed elevated binding in these regions together with more widespread binding in the temporal and parietal neocortex (Fig. 2b).

All five SV patients showed binding in the anterior temporal lobe, four showed binding in the ventromedial frontal cortex, and two also showed increased binding in the anterior hippocampus and amygdala. One of the three LV patients (patient 7) showed widespread [^18^F]THK5351 binding in the temporal, parietal and frontal cortices and in the precuneus bilaterally (Fig. 2c). The two other LV patients showed only restricted binding foci: patient 11 showed binding in the middle occipital gyrus bilaterally (Fig. 2d), and patient 9 showed minimally increased binding in the right middle temporal lobe and left premotor cortex.

#### Representative [^18^F]THK5351 SUVR images

**Fig. 3:**
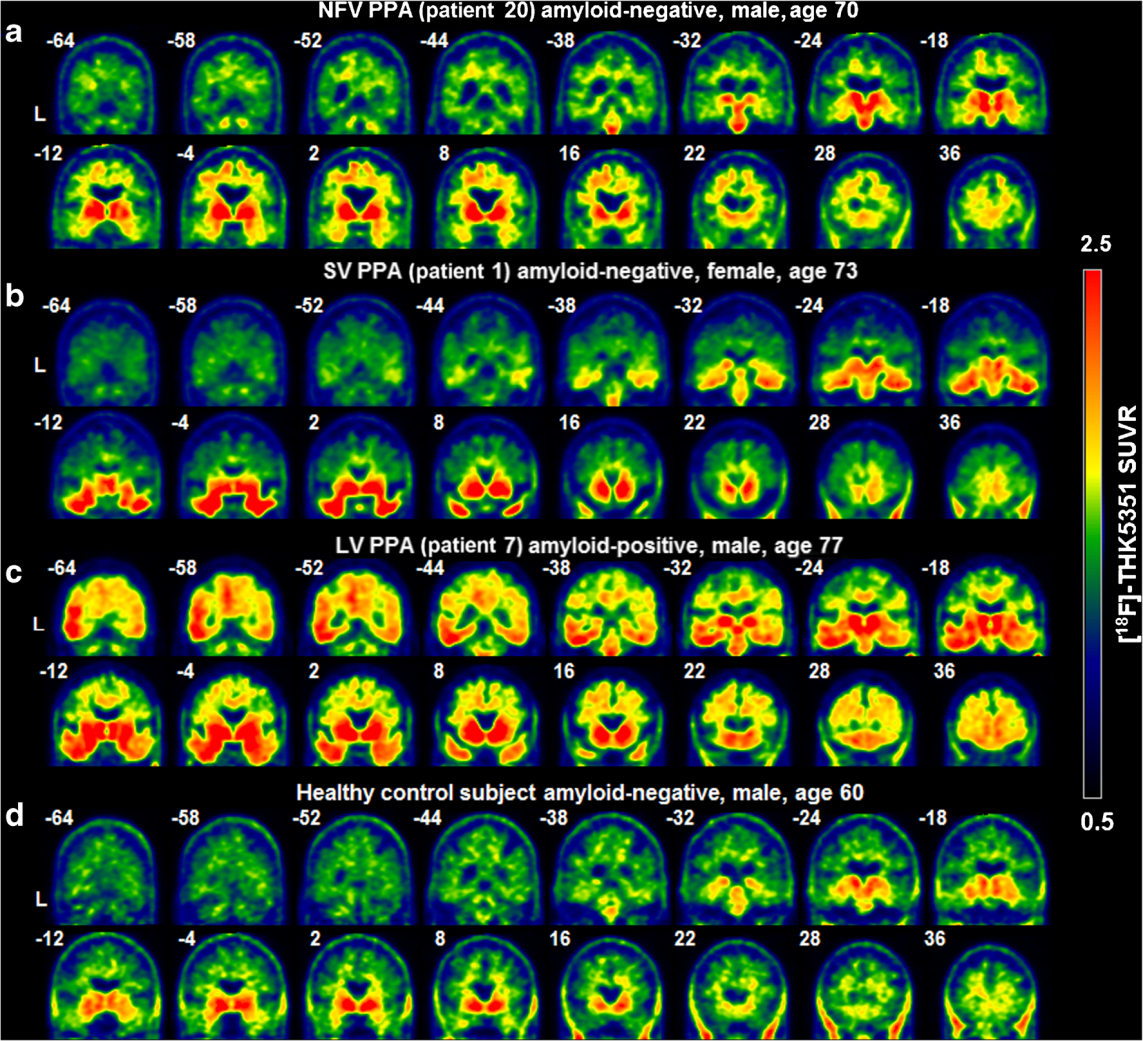
[^18^F]THK5351 SUVR images in representative individuals: **a** nonfluent variant (NFV) patient, **b** semantic variant (SV) patient, **c** logopenic (LV) patient, and **d** healthy control subject. The non-partial volume corrected SUVR intensity level is shown between 0.5 and 2.5. Patient numbers refer to Table 1

Figure 3 shows [^18^F]THK5351 SUVR images in representative patients in each group and a control subject. On visual inspection, the representative NFV patient (patient 20) showed increased [^18^F]THK5351 signal in the supplementary motor area, thalamus, basal ganglia and midbrain nuclei, with more pronounced binding in the left hemisphere (Fig. 3a). The representative SV patient (patient 1) showed highly increased [^18^F]THK5351 signal in the temporal lobes bilaterally and to a lesser degree in the ventromedial frontal cortex bilaterally (Fig. 3b). The representative LV patient (patient 7) showed increased [^18^F]THK5351 signal in the temporal lobes, temporo-occipital cortex, and the angular gyrus with additional involvement of the precuneus and frontal lobes (left > right; Fig. 3c). These representative PPA patients all showed binding in the basal ganglia, a pattern that was also seen in the control(s) (Fig. 3d).

### Secondary outcome analysis

#### [^18^F]THK5351 binding and degree of clinical deficit

**Fig. 4:**
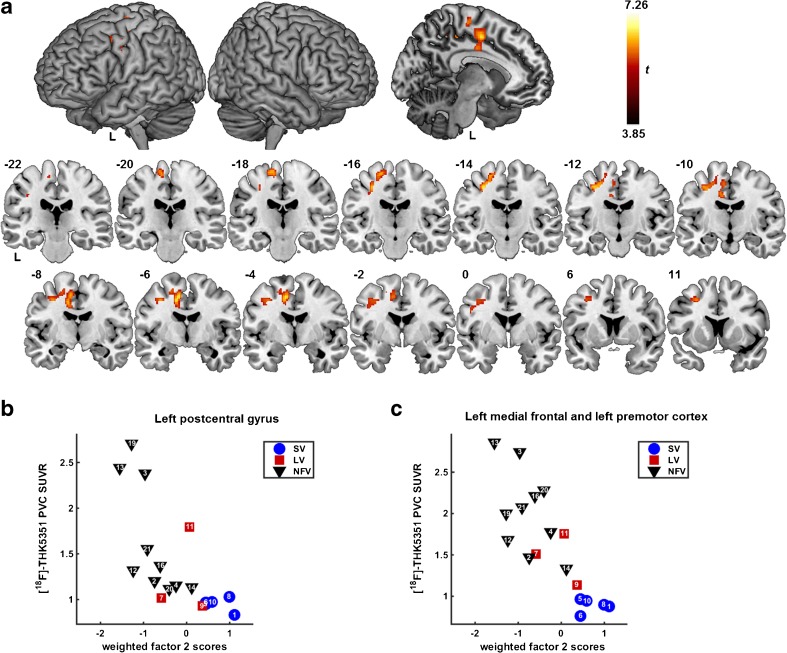
Whole-brain voxel-wise regression analysis between neurolinguistic factor scores representing agrammatism and apraxia of speech and [^18^F]THK5351 binding in PPA. **a** [^18^F]THK5351 binding in SUVR images with partial volume correction showing correlations with weighted factor 2 scores corrected for age, gender and education, depicted as a one-sided *t*-contrast on MNI template brain renderings and on coronal slices (brighter colour means higher *t* value). The significance threshold was set at voxel-level uncorrected *P* < 0.001 combined with cluster-level family-wise error-corrected threshold *P* < 0.05. **b**, **c** Scatterplots illustrating the correlations between the weighted factor scores of factor 2 and the extracted [^18^F]THK5351 SUVR values of the significant clusters in (**b**) the left postcentral gyrus and (**c**) the left supplementary motor area, cingulum and dorsal premotor cortex. The data points show patient numbers referring to Table 1

The factor analysis yielded two factors (Table 3). The first factor grouped measures of language comprehension and object recognition. The second factor grouped measures of agrammatism and motor speech impairment. The whole-brain voxel-wise regression analysis revealed a correlation between lower individual weighted factor scores on factor 2 and higher [^18^F]THK5351 binding in the left supplementary motor area, left dorsal premotor cortex, and left cingulum (Fig. 4; Table 4). No significant correlations were obtained between the individual weighted factor scores on factor 1 and [^18^F]THK5351 binding. In addition a voxel-wise linear regression analysis of the association between individual speech and language test scores and [^18^F]THK5351 binding was performed. Object recognition and naming were correlated with [^18^F]THK5351 binding in the anterior temporal lobes while sentence comprehension was correlated with binding in the left frontal operculum among other regions (Supplementary Table 2, Supplementary Fig. 2).

**Table 3:** Factor analysis of neurolinguistic test scores

	Factor 1	Factor 2
Eigenvalue	6.96	2.67
Variance explained (%)	49.7	19.1
Cumulative variance explained (%)	49.7	68.8
Pyramids and palm trees test	**0.952**	−0.063
BORB B easy	**0.844**	−0.132
AAT comprehension	**0.814**	0.423
BORB A hard	**0.791**	0.228
WEZT verb comprehension	**0.784**	0.281
Boston naming test	**0.742**	0.304
PALPA verbal associative-semantic test	**0.739**	0.214
Animal verbal fluency	0.646	0.525
WEZT sentence comprehension	0.334	**0.834**
PALPA pseudoword repetition	0.018	**0.795**
DIAS severity score	0.310	**0.755**
PALPA single-word repetition	−0.080	**0.743**
DIAS diadochokinesis total ratio	0.148	0.508
Coloured progressive matrices	0.334	0.394

Factor loadings greater than 0.7 are marked in bold

*AAT* Akense Afasie Test, *BORB* Birmingham Object Recognition Battery, *DIAS* Diagnostisch Instrument voor Apraxie van de Spraak (Diagnostic Instrument for Apraxia of Speech), *PALPA* Psycholinguistic Assessment of Language Processing in Aphasia, *WEZT* Werkwoorden En Zinnen Test (Verbs And Sentences Test)

**Table 4:** Peak coordinates of the whole-brain voxel-wise regression analysis between neurolinguistic factor scores representing agrammatism and apraxia of speech and PVC [^18^F]THK5351 binding in PPA

Cluster			Peak		MNI coordinates		
Name	*P* value	Size	*T*	*Z*	*x*	*y*	*z*
Left postcentral gyrus	0.007	349	7.26	4.51	−30	−34	44
			6.07	4.11	−38	−26	40
			4.83	3.59	−26	−42	36
Left medial frontal,	<0.001	644	6.01	4.09	−12	−6	46
Left premotor cortex			5.98	4.08	−30	−14	46
			5.59	3.92	−16	−18	66

The significance threshold was set at voxel-level uncorrected *P* < 0.001 combined with cluster-level family-wise error-corrected threshold *P* < 0.05, *T* *t* value, *Z* *z* value

#### Topography of [^18^F]THK5351 binding and atrophy

**Fig. 5:**
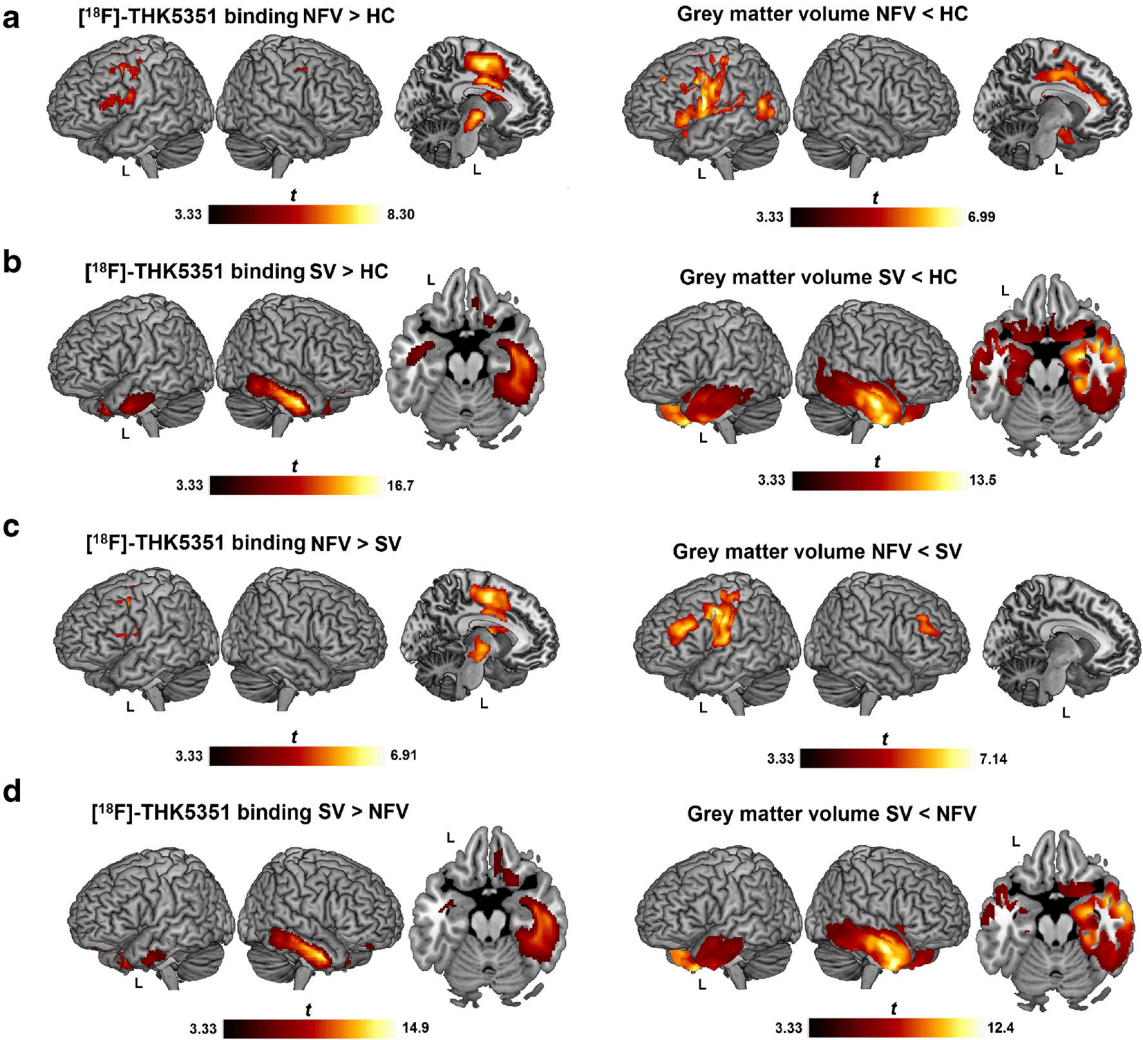
Topography of [^18^F]THK5351 binding and atrophy in PPA. Significantly increased [^18^F]THK5351 binding in SUVR images with partial volume correction and significant atrophy, compared using voxel-wise ANOVA, and depicted as a *t*-contrast overlaid on an MNI template brain renderings (brighter colour means higher *t* value). Nonfluent variant (NFV) PPA patient (**a**) and semantic variant (SV) PPA patient (**b**) compared with healthy controls (HC). **c** Higher binding and atrophy are apparent in the NFV patient compared with the SV patient. **d** Higher binding and atrophy are apparent in the SV patient compared with the NFV patient. The significance threshold was set at voxel-level uncorrected *P* < 0.001 combined with cluster-level family-wise error-corrected threshold *P* < 0.05

As shown in Fig. 5, [^18^F]THK5351 binding and MRI atrophy patterns were similar. The LV patients showed no significant atrophy at the pre-set significance threshold, most likely because of the small sample size and the heterogeneity in LV.

#### Differences in effect size between variants

**Fig. 6:**
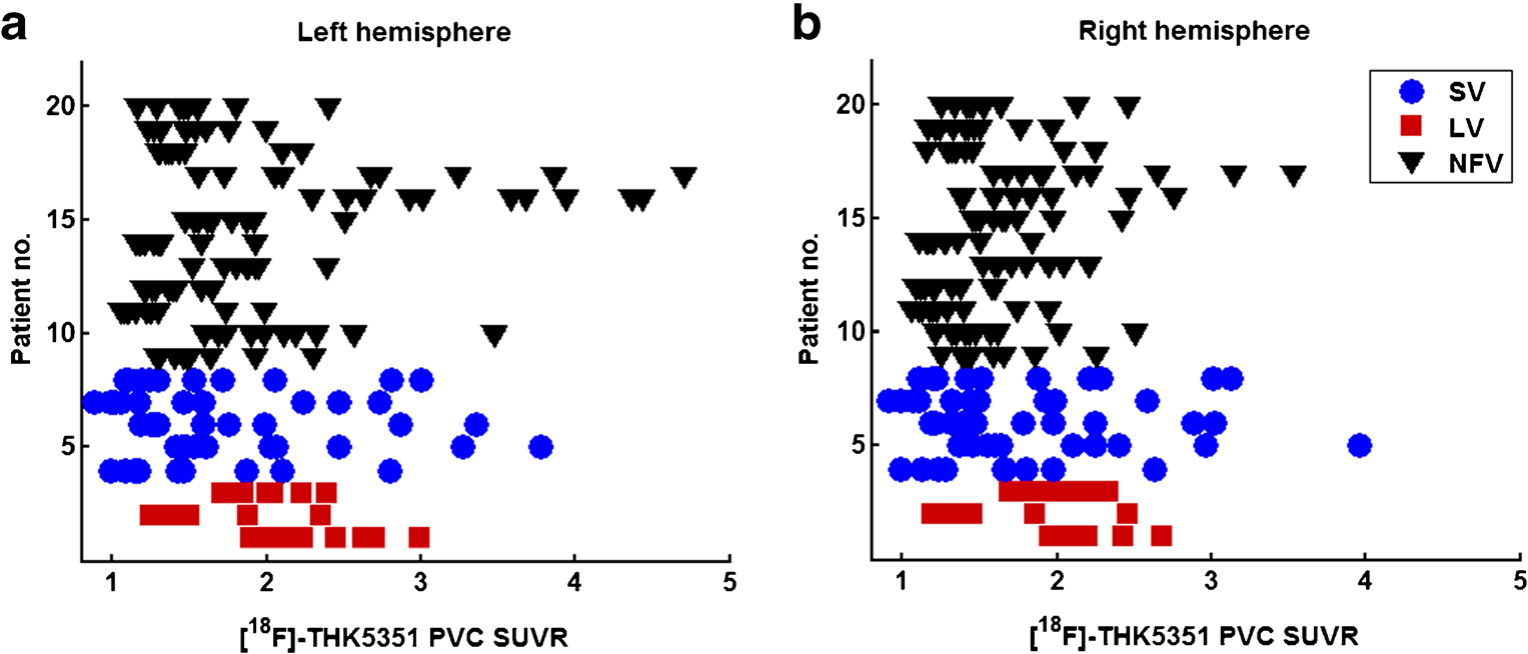
Effect size of [^18^F]THK5351 PET binding in individual patients with different PPA variants. The mean [^18^F]THK5351 SUVR with partial volume correction are plotted for the **a** left and **b** right hemispheres for individual patients with different PPA variants (*NFV* nonfluent variant, *SV* semantic variant, *LV* logopenic variant). Each *row* corresponds to a patient; the *colour* indicates the variant, and the individual *data points* correspond to the regional SUVR values of each of the ten ROIs per patient. The presentation of this figure is similar to that used by Josephs et al. [21]

Evaluation of the regional effect sizes on an individual basis showed the highest regional effect sizes (regional SUVR >4) in two NFV patients (patients 17 and 18; Fig. 6).

## Discussion

[^18^F]THK5351 imaging revealed characteristic patterns of binding in the three variants of PPA, with a topography that closely matched the anatomical distribution of the predicted underlying pathology for each variant [3, 4]. Elevated [^18^F]THK5351 binding in the left supplementary motor area and in the left postcentral gyrus correlated with clinical measures of agrammatism and speech production deficits. In SV patients, [^18^F]THK5351 binding was consistently increased in the anterior temporal cortex despite this being most commonly a TDP-43 proteinopathy.

NFV PPA is most frequently associated with a tauopathy (3R or 4R tau) [2–4]. Increased [^18^F]THK5351 binding was observed in all but one of the NFV patients with a topography that matched the known anatomical distribution of the lesions: elevated [^18^F]THK5351 binding in NFV patients compared with controls included the medial frontal, premotor and inferior frontal cortical regions as well as subcortical regions, namely the midbrain, thalamus and basal ganglia. There was also increased binding in the frontal subcortical white matter. These regions have increased vulnerability to FTLD tau [7, 41]. While the initial and most salient feature in NFV patients was language and speech impairment, a subset of NFV patients in this study showed mild clinical signs and symptoms that may be indicative of underlying PSP or CBD pathology, including right-sided extrapyramidal signs and vertical eye movement abnormalities (Supplementary Table 1). These patients may develop a PSP-like or CBD-like syndrome over time, similar to that described in patients with primary progressive apraxia of speech [42].

Two of the NFV patients were amyloid-positive. One had a focal pattern of uptake in the supplementary motor area and in the dorsal premotor cortex, and the other had widely distributed binding in the neocortical association zones of the left hemisphere, according to a pattern identical to that typically seen in AD, predominantly in the left hemisphere (Fig. 2). The THK5351 pattern in the former patient is in line with the notion that the distribution of tau PET abnormalities corresponds more closely to the clinical phenotype than amyloid PET [26], which was more isocortically elevated.

Elevated [^18^F]THK5351 binding in the left supplementary motor area was correlated with the degree of agrammatism and motor speech impairment. [^18^F]AV1451 binding in the supplementary motor area, dorsal premotor cortex and inferior frontal gyrus has been demonstrated previously in primary progressive apraxia of speech [43], a syndrome that most often shows underlying FTLD tauopathy on postmortem examination [5], and in patients with corticobasal syndrome who have apraxia of speech [44]. The current study is the first to show a quantitative relationship between speech apraxia measures and [^18^F]THK5351 binding. The supplementary motor area plays a crucial role in speech motor control [45]. Premotor cortical involvement has been linked to the severity of speech apraxia in MRI and [^18^F]FDG PET studies [46]. Damage to the white matter tract connecting the supplementary motor area to the inferior frontal gyrus (i.e. the aslant tract) affects the amount of distortion errors that NFV PPA patients make during spontaneous speech [45].

No correlation was observed between [^18^F]THK5351 binding and the first factor related to language comprehension. However, when the scores on the individual tests were correlated with [^18^F]THK5351 binding, correlations were seen between temporal cortical binding and the BNT, PPT and BORB scores. Based on MRI or [^18^F]FDG PET findings in SV patients [1], one would predict that in particular the left temporal lobe would be involved in comprehension deficits. SV patients indeed showed elevated [^18^F]THK5351 binding in this region compared with healthy controls and NFV PPA patients (Fig. 2). This finding is in accordance with the findings of previous studies of [^18^F]THK5351 binding [19, 24, 25] and [^18^F]AV1451 binding in SV patients [21–23]. The current cohort included only three LV patients. The pattern in LV was heterogeneous, in line with the heterogeneous composition of LV in general (for review see [6]). In one patient the pattern resembled that seen in typical AD (Fig. 2c) [17].

Overall, the outcome of the current study is similar to that in another recent study of the binding of [^18^F]AV1451 in PPA patients. The two tracers show topographical patterns that differentiate the three different PPA variants [21] and both studies showed focal binding in regions of predilection even in SV. There are also some differences between the two studies, which might be related to the tracer used or to the sample studied. At the individual level, the strongest regional effect sizes for [^18^F]AV1451 were obtained in LV patients [21], while in the current study the maximal regional effect sizes were highest in NFV patients and lowest in LV patients (Fig. 6). This could suggest differences between the tracers in their affinity for different types of tauopathy. Second, [^18^F]AV1451 binding in the frontal cortex was mostly restricted to the subcortical white matter, while [^18^F]THK5351 in the current study also showed neocortical binding in the regions typically affected in NFV, such as the supplementary motor area, precentral gyrus and inferior frontal gyrus (Fig. 1).

Two important issues regarding the first generation of tau PET tracers remain. First, high binding is systematically observed in the basal ganglia even in healthy controls for both [^18^F]THK5351 [19] and for [^18^F]AV1451 [10–12, 14, 47]. The [^18^F]AV1451 binding pattern might be attributed to neuromelanin in the basal ganglia and substantia nigra [11, 12, 14] or to affinity for monoamine oxidase-B (MAO-B) [48, 53]. Second, high binding is observed in SV, a disease that is most often associated with TDP-43 type C [2–4, 7]. As a first and obvious possibility, in vivo [^18^F]THK5351 may bind to the β-sheet conformation of TDP-43 type C aggregates. An [^18^F]THK5351 autoradiography study on human brain sections demonstrated higher affinity for the β-sheet conformation of tau than for fibrillary amyloid and no affinity for TDP-43 and α-synuclein [9]. An autoradiography study with a different tracer, PBB3, showed binding to α-synuclein if the aggregates were available in sufficiently high amounts [49], but this cannot yet be extrapolated to [^18^F]THK5351. Nevertheless, it remains possible that [^18^F]THK5351 binding in the anterior temporal lobe reflects binding to the β-sheet conformation of non-tau aggregates. As an alternative explanation, binding to MAO-B may play a role given the reactive astrogliosis that accompanies neurodegeneration in a stage-dependent manner [50] and the expression of MAO-B by reactive astrocytes [20, 51, 52]. Specific PET tracers for MAO-B have been developed, including [^11^C]deuterium-l-deprenyl [51]. In a postmortem binding study, deprenyl was shown to displace [^3^H]THK5351 binding by 40% in the frontal cortex and by 50% in the basal ganglia of AD patients [48]. In the AD dementia stage, neocortical binding of [^11^C]deuterium-l-deprenyl is not increased compared with that in controls [51]. Provided that the degree of reactive astrogliosis in neocortical regions of predilection is comparable between AD and PPA, reactive astrogliosis cannot account for the strong signal in SV in the temporal cortex. [^18^F]AV1451 also binds to MAO-A [53], and this could also be the case for [^18^F]THK5351. As a final possibility, these first-generation tau PET tracers may bind to a molecular target that is focally present in the regions of highest neurodegeneration, such as haem by-products or mineralized structures or a yet-undefined molecular target [14, 48]. Further work is needed to evaluate these different possibilities before the first-generation tau PET tracers can be considered valid biomarkers of the pathological processes underlying PPA.

The currently available CSF tau biomarkers are unable to positively detect underlying FTLD pathology [54] and are mainly useful for exclusion, i.e. to rule out AD as a cause [32]. [^18^F]THK5351 PET imaging might thus be of added value as a diagnostic measure in PPA patients as it provides a positive indication of underlying pathology, while [^18^F]FDG PET or MRI can only show regions of reduced metabolism or reduced grey matter, respectively. Moreover, [^18^F]THK5351 PET can show a positive signal early in the disease course, which would not be captured by [^18^F]FDG PET or MRI alone. We and others [19] have demonstrated that [^18^F]THK5351, despite its off-target binding, might be a valid option for measuring pathology associated with PPA. However, it is important that extensive preclinical characterization is performed including assessment of binding to tau maturation stages and to cell types affected before moving to clinical phase trials.

### Study limitations

No firm conclusions regarding the underlying type of tauopathy can be drawn as this study was limited by the lack of postmortem confirmation. Furthermore, the total number of PPA patients included was rather small, which is partly inherent in the relatively low prevalence of the syndrome [3]. The negative findings in LV patients are most likely due to the limited statistical power. Individual LV patients did show elevated signal, suggestive of binding to AD tauopathy. A drawback of using [^18^F]THK5351 PET is the nonspecific binding. Nonetheless, elevated [^18^F]THK5351 binding seen in the current study was highly focalized and colocalized with regions known to be associated with conditions in which a tauopathy is the underlying neuropathological cause [4, 7, 41].

### Conclusion

The primary outcome analysis showed that the [^18^F]THK5351 patterns were highly subtype-specific and in accordance with the predictions regarding the topography of the hallmark lesions in the different PPA variants. We were able to observe increases in signal colocalizing with areas of predilection in vivo. Elevated [^18^F]THK5351 binding in the left supplementary motor area correlated with clinical features, i.e. the degree of agrammatism and speech production deficit. The tracer may therefore serve as a topographically specific marker of neurodegeneration in patients with the different subtypes.

## Electronic supplementary material

ESM 1 (DOCX 1.61 mb)
