# Supplementary material for: Distinct [18F]THK5351 binding patterns in primary progressive aphasia variants
Source: Eur J Nucl Med Mol Imaging. 2018 Jun 26;45(13):2342–57. doi: 10.1007/s00259-018-4075-3 (PMC6208807; doi:10.1007/s00259-018-4075-3)
Supplement: Supplementary file 1 — (DOCX 1.61 mb) [file 259_2018_4075_MOESM1_ESM.docx]

**Distinct [^18^F]-THK5351** **binding patterns in primary progressive aphasia variants**

**European Journal of Nuclear Medicine and Molecular Imaging**

Jolien Schaeverbeke^1,2^, Charlotte Evenepoel^1,2^, Lieven Declercq^10,11^, Silvy Gabel^1,2^, Karen Meersmans^1^, Rose Bruffaerts^1,3^, Kate Adamczuk^1,2^, Eva Dries^3^, Karen Van Bouwel^3^, Anne Sieben^4,5,6^, Yolande Pijnenburg^7,8^, Ronald Peeters^9^, Guy Bormans^10,11^, Koen Van Laere^2,11^, Michel Koole^11^, Patrick Dupont^1,2^, Rik Vandenberghe^1,2,3^

^1^Laboratory for Cognitive Neurology, Department of Neurosciences, KU Leuven, Herestraat 49, 3000 Leuven, Belgium; ^2^Alzheimer Research Centre KU Leuven, Leuven research Institute for Neuroscience & Disease, KU Leuven, Herestraat 49, 3000 Leuven, Belgium; ^3^Neurology Department, University Hospitals Leuven, Herestraat 49, 3000 Leuven, Belgium; ^4^Center for Molecular Neurology, VIB, Universiteitsplein 1, 2610 Antwerp, Belgium; ^5^Institute Born-Bunge, University of Antwerp, Universiteitsplein 1, 2610 Antwerp, Belgium; ^6^Neurodegenerative Brain Diseases Group, Center for Molecular Neurology, VIB, Universiteitsplein 1, 2610 Antwerp, Belgium; ^7^Institute Born-Bunge, Neuropathology and Laboratory of Neurochemistry and Behavior, University of Antwerp, Universiteitsplein 1, 2610 Antwerp, Belgium; ^8^Neurology Department, University Hospital Ghent, Corneel Heymanslaan 10, 9000 Ghent, Belgium; ^9^Old Age Psychiatry Department, GGZinGeest, Van Hilligaertstraat 21, 1072 JX Amsterdam, The Netherlands; ^10^Laboratory of Radiopharmaceutical Research, KU Leuven, Herestraat 49, 3000 Leuven, Belgium; ^11^Nuclear Medicine and Molecular Imaging, University Hospitals Leuven, Herestraat 49, 3000 Leuven, Belgium.

Corresponding author: Rik Vandenberghe, MD, PhD Neurology Department, University Hospitals Leuven, Herestraat 49 - box 7003 Leuven, Belgium, 3000 Phone: +3216344280, Fax: +3216344285 rik.vandenberghe@uz.kuleuven.ac.be.

- 1. **Amyloid biomarker measurement and analysis**

[^11^C]-PIB PET was obtained within 2-182 days from the [^18^F]-THK5351 PET scan (mean: 82 days in controls; mean: 19 days in PPA patients). [^11^C]-PIB PET scans were acquired on a GE Signa 3T PET/MR scanner (GE Healthcare, Chicago, USA) operating in 3D mode to estimate amyloid burden in eight patients and 14 healthy control subjects (data of 6 control subjects were not included due to technical issues). [^11^C]-PIB was injected intravenously as a bolus in an antecubital vein (controls: mean dose=267.8 MBq, range: 197.5-364.9 MBq; patients: mean dose=270.5 MBq, range: 230.6-316.4 MBq). Dynamic [^11^C]-PIB images were acquired during a 70-min period and reconstructed with an atlas-based attenuation correction method using the manufacturer's software. Two patients received a 30-min [^11^C]-PIB PET scan in a clinical context between 40 and 70 min *post* injection on the same Siemens Biograph PET/CT scanner used for [^18^F]-THK5351 PET. For the purpose of comparing [^11^C]-PIB binding in these two patients, [^11^C]-PIB scans of in 19 older age- and gender-matched amyloid-negative cognitively intact control subjects, acquired on the same scanner were used. Processing of [^11^C]-PIB PET scans was done in SPM12 using the same MRI-based method as described for [^18^F]-THK5351. The mean [^11^C]-PIB PET SUVR value was calculated in a neocortical composite region [33] and considered positive if this value was significantly elevated compared to healthy controls based on a modified *t-*test (α<0.05) [39]. This test was performed separately for the eight and two patients respectively acquired on a PET/MR and PET/CT scanner with separate normative groups each.

- 1. **Volumetric MRI acquisition and analysis**

A high resolution T_1_-weighted structural MRI scan was acquired on a 3 Tesla Philips Achieva scanner equipped with a 32-channel head coil (Philips, Best, The Netherlands) using a 3D turbo field echo sequence (coronal inversion recovery prepared 3D gradient-echo images, inversion time (TI) 900 ms, shot interval = 3000 ms, echo time (TE) = 4.6 ms, repetition time (TR) = 9.6 ms, flip angle 8 degrees, field of view (FoV)= 250 x 250 mm, 182 slices, slice thickness = 1.2 mm, voxel size = 0.98x1.2x0.98 mm^3^).

**Supplementary table 1. Clinical signs and symptoms in PPA**

|  | **Nonfluent variant** | | | | | | | | | | | | **Semantic variant** | | | | | **Logopenic variant** | | |
| --- | --- | --- | --- | --- | --- | --- | --- | --- | --- | --- | --- | --- | --- | --- | --- | --- | --- | --- | --- | --- |
|  | 2 | 3 | 4 | 12 | 13 | 14 | 16 | 17 | 18 | 19 | 20 | 21 | 1 | 5 | 6 | 8 | 10 | 7 | 9 | 11 |
| **Signs and symptoms** |  |  |  |  |  |  |  |  |  |  |  |  |  |  |  |  |  |  |  |  |
| Hypomimetic facies | - | - | + | + | - | - | - | - | - | - | - | - | - | - | - | - | - | - | - | - |
| Dysarthria | - | - | + | + | - | - | - | - | - | - | - | + | - | - | - | - | - | - | - | - |
| Right-sided limb dystonia | - | - | + | - | - | - | - | - | - | - | - | - | - | - | - | - | - | - | - | - |
| Right-sided extrapiramidal signs | - | - | + | + | + | + | - | - | - | + | + | - | - | - | - | - | - | - | - | - |
| Alien limb | - | - | - | + | - | - | - | - | - | - | - | - | - | - | - | - | - | - | - | - |
| Nuchal rigidity | - | - | + | - | + | - | - | - | - | - | - | + | - | - | - | - | - | - | - | - |
| Reduced postural reflexes | - | - | + | - | - | - | - | - | - | - | - | - | - | - | - | - | - | - | - | - |
| Falls | + | - | + | + | - | - | - | - | - | - | - | - | - | - | - | - | - | - | - | - |
| Tremor | - | - | - | + | + | - | - | - | - | - | - | - | - | - | - | - | - | - | - | - |
| Myoclonus | - | - | - | - | - | - | - | - | - | - | - | - | - | - | - | - | - | - | - | - |
| Vertical Gaze slowing or palsy | - | - | + | + | + | + | + | - | - | - | + | + | - | - | - | - | - | - | - | - |
| Decrease in vertical optokinetic nystagmus | + | - | + | + | + | + | + | - | - | - | + | + | - | - | - | - | - | - | - | - |
| Piramidal signs | - | + | - | + | - | - | - | - | - | - | - | - | - | - | - | - | - | - | - | - |
| Dysphagia | + | - | + | - | - | - | - | - | - | - | - | - | - | - | - | - | - | - | - | - |
| Pseudobulbar affect | + | - | - | - | - | - | - | - | - | - | - | - | - | - | - | - | - | - | - | - |
| Ideomotor apraxia | - | - | - | - | - | - | - | - | - | - | - | - | - | - | - | - | - | - | - | - |
| Apraxia of eyelid closure | - | - | - | - | - | - | - | - | - | - | - | - | - | - | - | - | - | - | - | - |

**Supplementary table 2. Peak coordinates of the whole-brain voxel-wise regression analysis between individual language tests used in the factor analysis and PVC [^18^F]-THK5351 binding.**

| Language test | Cluster name | cluster | cluster | peak | peak |  | MNI coordinates |  |
| --- | --- | --- | --- | --- | --- | --- | --- | --- |
|  |  | ***P*** | **size** | ***T*** | ***Z*** | **x** | **y** | **z** |
| PPT | L inferior temporal g. | 0.043 | 255 | 6.63 | 4.39 | -38 | -2 | -28 |
|  | R inferior temporal g. | 0.002 | 516 | 4.85 | 3.65 | 42 | -4 | -36 |
|  |  |  |  | 4.12 | 3.28 | 52 | -16 | -24 |
| BORB B easy | R fusiform g. | <0.001 | 1374 | 4.90 | 3.68 | 38 | -4 | -36 |
|  |  |  |  | 4.71 | 3.59 | 38 | -40 | -22 |
|  |  |  |  | 4.65 | 3.56 | 52 | -28 | -12 |
| BNT | L inferior temporal g. | <0.001 | 1266 | 5.69 | 4.03 | -46 | -38 | -16 |
|  |  |  |  | 5.03 | 3.74 | -48 | -10 | -32 |
|  |  |  |  | 4.72 | 3.59 | -50 | -48 | -4 |
| WEZT | L frontal | 0.007 | 346 | 9.02 | 5.00 | -46 | -2 | 18 |
| sentence | operculum |  |  | 4.96 | 3.65 | -36 | 10 | 16 |
| comprehension ^a^ |  |  |  | 4.72 | 3.54 | -22 | 18 | 8 |
|  | L precuneus | <0.001 | 625 | 6.22 | 4.16 | -24 | -72 | 22 |
|  |  |  |  | 5.83 | 4.02 | -14 | -56 | 44 |
|  |  |  |  | 5.40 | 3.84 | -14 | -62 | 26 |
|  | L middle | 0.029 | 251 | 5.91 | 4.05 | -8 | -4 | 48 |
|  | cingulum |  |  | 4.89 | 3.62 | -8 | -8 | 38 |
|  | L SMA |  |  | 4.79 | 3.57 | -10 | 12 | 48 |
| DIAS | R precuneus | 0.009 | 363 | 5.49 | 3.95 | 12 | -66 | 40 |
| diadocho- |  |  |  | 4.91 | 3.68 | 12 | -56 | 44 |
| kinesis |  |  |  | 4.42 | 3.44 | 18 | -70 | 32 |

| Abbreviations: BNT: Boston Naming Test; BORB: Birmingham Object Recognition Battery; DIAS: Diagnostisch Instrument voor Apraxie van de Spraak (Diagnostic Instrument for Apraxia of Speech); g.: gyrus; L: left; R: right; SMA: supplementary motor area; WEZT: Werkwoorden En Zinnen Test (Verbs And Sentences Test). The significance threshold was set at voxel-level uncorrected *P*<0.001 combined with cluster-level family wise error (FWE)-corrected threshold *P*<0.05. ^a^Correlation based on 18 PPA patients |
| --- |

**Supplementary table 3. Peak coordinates of whole-brain voxel-wise ANOVA with grey matter volume.**

| Cluster name | cluster | cluster | peak | peak |  | MNI coordinates |  |
| --- | --- | --- | --- | --- | --- | --- | --- |
|  | ***P*** | **size** | ***T*** | ***Z*** | **x** | **y** | **z** |
| Grey matter volume NFV < healthy controls | | | | | | | |
| left pars opercularis, | <0.001 | 9444 | 6.99 | 5.52 | -55.5 | 7.5 | 15 |
| insula, |  |  | 6.49 | 5.25 | -51 | 4.5 | 7.5 |
| premotor cortex, |  |  | 6.22 | 5.09 | -40.5 | 7.5 | 36 |
| precentral gyrus, | 0.01 | 1416 | 6.09 | 5.02 | -48 | -60 | 10.5 |
| supplementary motor area, |  |  | 4.82 | 4.20 | -45 | -75 | 3 |
| cingulum, |  |  | 3.64 | 3.34 | -52.5 | -55.5 | -4.5 |
| pars triangularis, | 0.008 | 1596 | 6.00 | 4.97 | -21 | -7.5 | 55.5 |
| and hippocampus |  |  | 5.38 | 4.58 | -21 | 39 | 37.5 |
|  |  |  | 5.35 | 4.56 | -28.5 | -13.5 | 49.5 |
|  | 0.001 | 2392 | 5.19 | 4.46 | -4.5 | -6 | 46.5 |
|  |  |  | 4.86 | 4.24 | -7.5 | 6 | 43.5 |
|  |  |  | 4.71 | 4.13 | -7.5 | 43.5 | 15 |
| Grey matter volume SV < healthy controls | | | | | | | |
| temporal lobes, | <0.001 | 44094 | 13.45 | Inf | 58.5 | 3 | -27 |
| ventromedial frontal cortex |  |  | 12.53 | 7.72 | 30 | -6 | -21 |
|  |  |  | 12.34 | 7.67 | 45 | 7.5 | -46.5 |
| Grey matter volume NFV < SV | | | | | | | |
| left premotor cortex, | <0.001 | 4324 | 7.14 | 5.60 | -43.5 | 1.5 | 42 |
|  |  |  | 6.40 | 5.20 | -52.5 | -4.5 | 31.5 |
| dorsolateral prefrontal cortex |  |  | 6.13 | 5.04 | -42 | -10.5 | 33 |
|  | 0.008 | 1591 | 5.97 | 4.94 | -36 | 36 | 24 |
|  |  |  | 5.89 | 4.90 | -43.5 | 42 | 21 |
|  |  |  | 5.78 | 4.83 | -42 | 30 | 27 |
|  | 0.045 | 1015 | 5.19 | 4.45 | 27 | 36 | 34.5 |
|  |  |  | 4.82 | 4.20 | 34.5 | 43.5 | 22.5 |
|  |  |  | 3.91 | 3.55 | 33 | 54 | 19.5 |
| Grey matter volume SV < NFV | | | | | | | |
| temporal lobes, | <0.001 | 22982 | 12.39 | 7.68 | 45 | 7.5 | -46.5 |
| ventromedial frontal cortex |  |  | 12.21 | 7.63 | 58.5 | 3 | -27 |
|  |  |  | 10.81 | 7.17 | 37.5 | -24 | -24 |
|  | <0.001 | 7326 | 6.73 | 5.38 | -37.5 | 3 | -45 |
|  |  |  | 6.53 | 5.27 | -24 | 1.5 | -48 |
|  |  |  | 5.87 | 4.88 | -22.5 | 13.5 | -40.5 |

Abbreviations: NFV: nonfluent variant; SV: semantic variant. The significance threshold was set at voxel-level uncorrected *P*<0.001 combined with cluster-level family wise error (FWE)-corrected threshold *P*<0.05


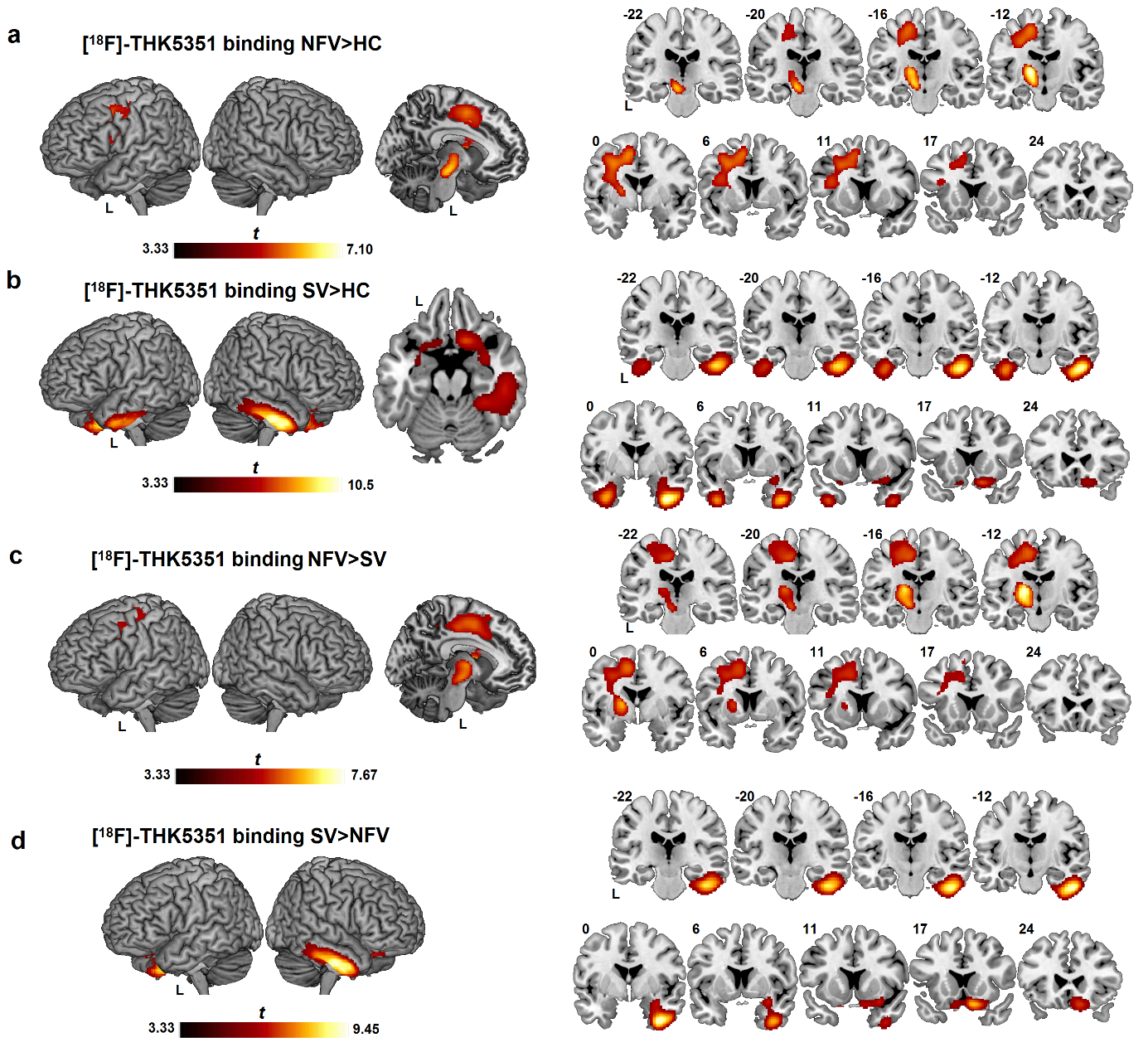


**Supplementary Fig. 1 Non-partial volume corrected group-wise comparisons of [^18^F]-THK5351** **binding**

Significantly elevated [^18^F]-THK5351 binding on non-partial volume corrected SUVR images, statistically contrasted using voxel-wise ANOVA, is depicted by a one-sided *t*-contrast overlaid on an MNI template brain rendering and on coronal slices. **(a)** Higher binding in nonfluent variant (NFV) PPA, **(b)** semantic variant (SV) PPA, compared to healthy controls (HC). **(c)** Higher binding in NFV compared to SV, **(d)** higher binding in SV compared to NFV. The significance threshold was set at voxel-level uncorrected *P*<0.001 combined with cluster-level family wise error (FWE)-corrected threshold *P*<0.05


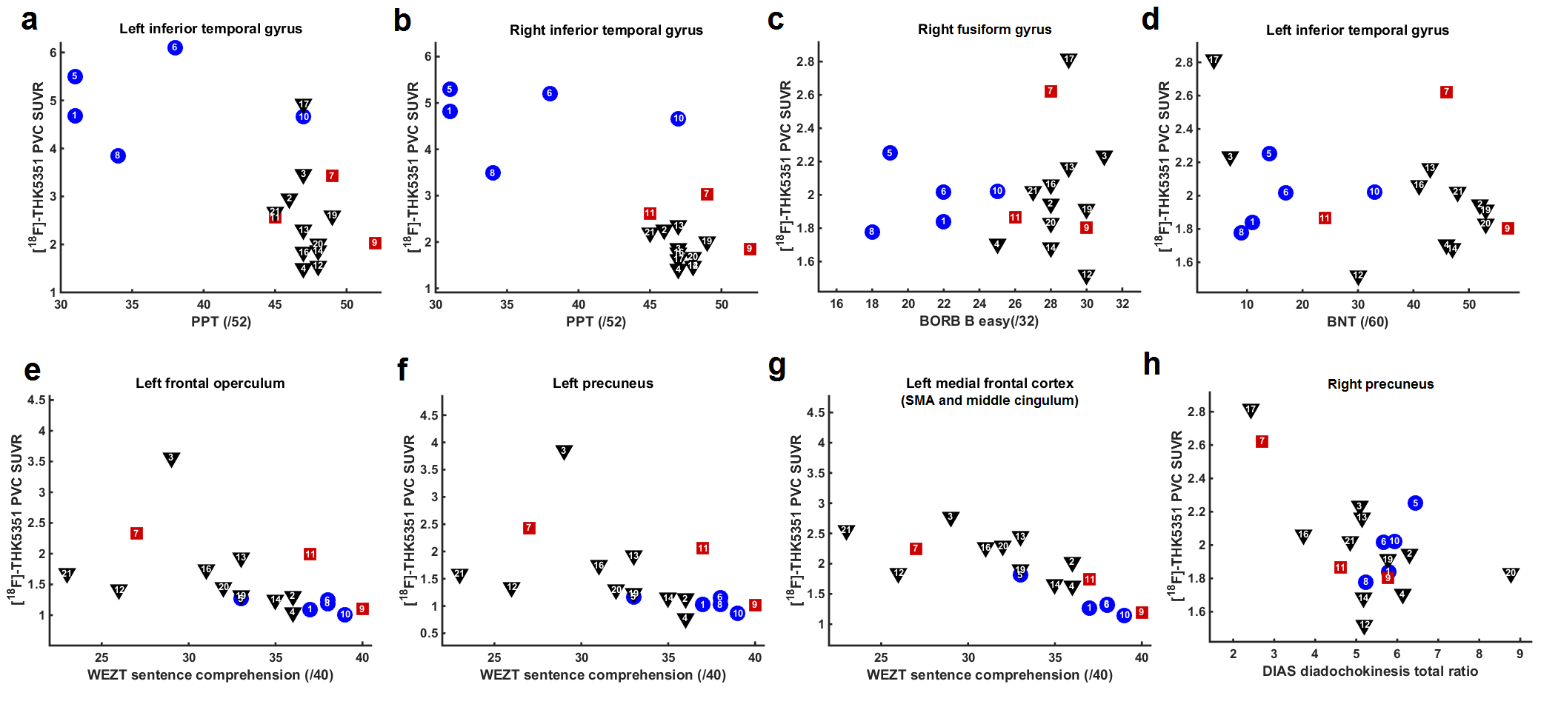


**Supplementary Fig. 2 Correlations between [^18^F]-THK5351** **binding and individual language tests**

The scatterplots illustrate the results from the whole-brain voxel-wise correlation between partial volume corrected [^18^F]-THK5351 SUVR images binding and the individual language test scores, corrected for age, gender and education. Significant clusters were extracted from SPM and mean [^18^F]-THK5351 uptake was calculated in each cluster and plotted against test scores for **(a)-(b)** Pyramids and Palm trees Test (PPT) for written words and pictures, **(c)** Birmingham Object Recognition Battery (BORB) B easy, **(d)** Boston Naming Test (BNT), **(e)-(g)** Werkwoorden En Zinnen Test (Verbs And Sentences Test) (WEZT) sentence comprehension, **(h)** Diagnostisch Instrument voor Apraxie van de Spraak (Diagnostic Instrument for Apraxia of Speech) (DIAS) diadochokinesis total ratio. Other language tests did not show a significant correlation at the whole-brain level. The significance threshold was set at voxel-level uncorrected *P*<0.001 combined with cluster-level family wise error (FWE)-corrected threshold *P*<0.05. Case numbers refer to Table 1
